# Management of critically located brain metastases in patients with precluded survival using customised double-dose prescription-based, adaptive accelerated staged radiosurgery: a long-term retrospective analysis

**DOI:** 10.1186/s13014-025-02692-x

**Published:** 2025-08-01

**Authors:** G. Sinclair, H. Martin, C.M. Allison, M.A. Hatiboglu, H. Speckter, A. Fytagoridis

**Affiliations:** 1 Department of Neuroradiology, Karolinska University Hospital, Stockholm, Sweden; 2 Department of Neurosurgery, Karolinska University Hospital, Stockholm, Sweden; 3 Department of Neurosurgery, Bezmialem Vakif University Hospital, Istanbul, Turkey; 4 Department of Neurosurgery, Royal Victoria Infirmary, Newcastle-upon- Tyne, UK; 5 Department of Oncology, University Hospital Southampton NHS Foundation Trust, Southampton, UK; 6 Centro Gamma Knife Dominicano, CEDIMAT, Santo Domingo, Dominican Republic

**Keywords:** Brain metastases, Radiosurgery, Gamma Knife, Linear accelerator, Prescription dose, Hypofractionated radiotherapy, Staged radiosurgery

## Abstract

**Background:**

Patients with brain metastases face a poor prognosis when deemed not to be suitable for onco-surgical intervention. This feasibility study analyses the outcome of adaptive radiosurgery applied in customised settings to a group of patients with brain metastases, where no other form of treatment was deemed safe and/or feasible.

**Methods:**

29 patients with 35 brain metastases deemed not to be candidates for surgery, radiotherapy or systemic treatment were treated with MRI-guided adaptive Gamma Knife radiosurgery in 3 separate sessions with a 72-hour interval. Customised synchronous double-dose prescriptions were systematically utilised at each session. Estimated survival at pre-treatment was fewer than 4 weeks due to the targeted intracranial lesions. A retrospective analysis was conducted on the whole cohort, with particular emphasis on those surviving 12 months and beyond.

**Results:**

The median overall survival was 7.3 months, with a follow-up of 7.5 years. Survival at 6, 12, 24, 36, 48 and 60 months was 62%, 41%, 17%, 14%, 10% and 7%, respectively. Local tumour control (LTC) at 1 year was 75%. 4 patients developed local recurrence late on follow-up, with a survival ranging between 12 and 40 months. 2 patients were alive at the time of submission (115- and 117-months post-treatment) with no sequelae; the remainder succumbed to general disease progression, with neurologic death being avoided throughout the whole cohort. Adverse radiation effects (ARE) were reported in 5 patients, of which 4 remained asymptomatic throughout follow-up.

**Conclusions:**

Based on the results of this first retrospective study, adaptive radiosurgery in double-dose prescription settings provided acceptable rates of LTC and ARE despite the underlying accelerated timeline, ultimately preventing neurologic death in a group of patients with an extremely poor prognosis. Prospective studies involving a larger number of patients with homogenous histology are warranted to validate the results of this study and optimise the technique.

**Supplementary Information:**

The online version contains supplementary material available at 10.1186/s13014-025-02692-x.

## Introduction

From a historical standpoint, Gamma Knife Radiosurgery (GKRS) was initially limited to single fraction treatments of small to medium-sized brain metastases (BM) until the development of hypofractionated schedules, which allowed the inclusion of larger lesions. However, despite remarkable technical advances in the field of radiosurgery, the risk of adverse radiation effects remains a concern when treating BM, particularly those with large volumes in the classical term (> 8-10cm3 / >2–3 cm) and/or eloquent location beyond size-thresholds [1–10]. Furthermore, despite providing excellent rates of local tumour control, median survival following GKRS of BM rarely exceeds 24 months, mainly due to extracranial disease progression [1]. In this context, best outcomes are often reported in patients retaining a WHO performance status of 0–1 (Karnofsky Performance Status > 70) and stable extracranial disease / best Recursive Partitioning Analysis (RPA) class [11–15]. Moreover, dose-volume constraints associated with previous radiotherapy and/or critical location (such as brainstem proximity) often prohibit key supplementary focal therapy regardless of extracranial disease status. Consequently, radiosurgery is often excluded in those deemed not to be candidates for repeat neurosurgical and/or oncological intervention in the CNS, increasing the risk of neurological death.

It is well-established that delivering a lethal dose to the tumour bed whilst retaining tolerable doses to surrounding healthy tissues can be attained by increasing the number of fractions. As a result, a series of hypofractionation protocols have gradually evolved over the last few decades, mostly based on Quantec-estimates [16, 17] and local task force policies; however, dose prescriptions are seldom customised beyond determined dose-volume surrogate estimates (usually V_10 − 12 Gy,_ for single fraction schedules and V_18 − 21 Gy_ in hypofractionated settings) [1, 3, 18–25]. Aiming to modulate and optimise the outcome of stereotactic radiation treatments within this entangled framework, a shift towards bespoke dose prescriptions balancing biomathematical estimates with netted clinical variables is warranted, particularly in high-risk patients. With this in mind, we conducted a feasibility study applying adaptive radiosurgery based on two customised simultaneous dose prescriptions on a cohort of patients with BM (*n* = 29), who were not candidates for any standard treatment (including single fraction radiosurgery) and whose expected survival due to the targeted lesion was fewer than 4 weeks at multidisciplinary team assessment. The accelerated timeline of delivery contributed to the novel qualities of the procedure.

Previously published data retrospectively analysing the subacute effects of treatment on 28 patients treated with the technique, demonstrated a volume size reduction of approximately 10% during the week of treatment and 50% on MRI at 4 weeks [4–7, 26]. To validate the latter results as well as the overall effects in the long term, continual follow-up was deemed necessary. Due to the inherent poor prognosis, particular focus was placed on those patients surviving at least 12 months post-treatment. Total follow-up time was concluded to be at least 5 years, when possible. To our knowledge, studies involving radiation dose customisation within the CNS remain scarce. Furthermore, studies on hypofractionated/staged GKRS remain limited in comparison to linear accelerator (LINAC)-based therapies, particularly in time-accelerated, adaptive setups [8]. Likewise, studies on post-radiosurgery outcomes have a limited timescale, generally not exceeding 2 years.

## Materials and methods

### Patient selection

Between 2013 and 2017, 29 patients with 35 BM were treated in next-to acute settings with adaptive staged GKRS at Karolinska University Hospital (Stockholm, Sweden), applying an accelerated timeline of a 72-hour interval, a procedure previously referred to as “Rapid Rescue Radiosurgery” [26]. Regional and hospital ethical board approval was obtained as per local hospital policy. Before treatment, patients were assessed by a multidisciplinary team consisting of staff from Neurosurgery, Gamma-Knife surgery, Neuro-Oncology, Neuroradiology, Neuropathology, Neurology and Medical Physics, where required.

#### Inclusion criteria for treatment

1. Patients with oligometastatic brain disease (1–3 BM).
2. KPS 70–100 (WHO performance status 0–2)/RPA 1–2 (unless compassionate treatment on patients with KPS < 70 / RPA 3 to salvage the local neurofunction and avoid neurologic death).
3. Not a candidate for surgery by multidisciplinary team assessment*.
4. Not a candidate for any form of radiotherapy by multidisciplinary assessment (including single-session gamma knife radiosurgery due to local constraints such as tumour volume, previous radiation of critical areas or adjacent organs at risk)*.
5. Not a candidate for systemic treatment with CNS-penetration by multidisciplinary assessment*.
6. Possibility of benefitting from systemic treatment if accepted for adaptive accelerated staged GKRS (at the oncologist’s discretion).

(*Neurosurgery, Gamma Knife surgery, Neuroradiology, Neuro-oncology, Neuropathology with the support of Medical Physics when necessary)

#### Exclusion criteria

1. Multiple synchronous BM (4 or more), unless belated decision for compassionate treatment based on planning MRI and clinical status.
2. KPS < 70/RPA 3, unless compassionate treatment as per point 2 above.

Expected neurologic death for all patients was estimated to be 4 weeks or less, hence the decision for a shorter interfraction timeline. As per institutional experience, target ‘largeness’ was determined by balancing volumetric estimates with regional functionality; in general, lesions with a volume > 8cm3 in the non-critical areas and those < 8cm3 in the eloquent brain were assessed as ‘large’ [4–8], hence suitable for adaptive accelerated, staged GKRS. To follow and validate previous publications on this procedure [4–7, 26], a retrospective analysis focusing on survival, LTC (Local tumour control) and ARE (Adverse radiation effects) at long-term was conducted on all 29 patients, using relevant clinical, radiological, and treatment planning data, under a follow-up period of at least 5 years (if possible). Considering the intrinsically poor prognosis at multidisciplinary outcome (adaptive accelerated staged GKRS vs. best supportive care), distinct attention was made to those surviving at least 12 months post-treatment. Consequently, 2 sub-cohorts were analysed: those surviving less than one year and those surviving longer, with particular interest placed on the latter. For quality assurance, findings were compared to similar studies in the literature.

### Treatment protocol

The Elekta Gamma Knife Perfexion was utilised for this study. All BM were treated using three separate sessions (GKRS 1–3), with an approximate 72-hour interval as an inpatient procedure [26–28]. Optimal cranium immobilisation was achieved by mounting the Leksell Coordinate Frame G under local anaesthetic prior to each fraction (GKRS 1–3: three separate frame applications over a period of one week). For image-guided treatment purposes, patients underwent a stereotactic MRI on GE Discovery MR450 1.5T (GE Medical Systems), with the frame on prior to each GKRS. Stereotactic MRI sequences included: (i) unenhanced 4 mm sagittal T1 SE, (ii) post-Dotarem 0.2 mmol/kg gadolinium contrast-enhanced 3D T1 FSPGR, (iii) 3 mm axial T1 FSE, and (iv) 4 mm axial T2 propeller. Upon target identification on contrast-enhanced T1 and T2-weighted MR series, no margins were applied to the GTV (Gross Tumour Volume). The ensuing treatment planning, which included a baseline plan for GKRS 1 and subsequent re-planning of GKRS 2–3 utilising the LGP (Leksell Gamma Knife Planning)-system, was structured on two simultaneous dose prescriptions, applied on the entire cohort:

#### First prescrition: Peripheral prescription dose (PPD) for sparing of healthy perilesional tissue (Gy and % isodose)

In accordance with Brenner et al. [29] and current institutional experience, PPD prescriptions were conceived using the LQ (linear quadratic) model and consequent BED (biological effective dose) / EQD2-based iso-conversions [30–32], historically applied to hypofractionated schedules of up to 10 Gy per fraction [29]. An α/β ratio of 2 was utilised in all calculations. The applied LQ-based BED formula can be found in the appendix list (Appendix 5) [30–32].

**Variables defining PPD at GKRS 1**:

- BED estimates from previous LINAC-based radiotherapy (such as WBRT or stereotactic hypofractionated treatments) were considered and subtracted in the calculus of protective BED estimates.
- Distinction was made between radio-resistant (renal cell carcinoma, malignant melanoma, mesothelioma, and colorectal adenocarcinomas) and radiosensitive histology (lung, thymus, ovarian and breast cancer) [33, 34]. For radio-resistant lesions [35], the PPD was to be increased by 1–2 Gy compared to radiosensitive histology.
- Oedema was not considered to be a precluding factor to adaptive accelerated staged GKRS. As a result, regardless of extension, perilesional oedema alone did not influence the choice of dose prescription at any stage of treatment.
- Once integrating the above to location and dose-tolerance data, two subgroups were identified for the purpose of PPD definition:
  - 1. Brainstem location: brainstem tolerance in terms of EQD2 (equivalent dose in 2 Gy) was set at 54 Gy for the entire axis and 60 Gy for up to 1/3 (about 8-10 cc) of the brainstem volume [36].
  - 2. Non-brainstem location: Dose tolerance was set at 50–60 Gy (EQD2) in patients with lesions located in highly functional areas and/or near critical organs (including motor-sensor cortex, neighbouring brainstem, thalamus, optic pathways, temporal lobe); in less critical areas (such as the peripheral cerebellum and parietal regionality), we assessed dose tolerance to be max 100 Gy (EQD2) [37, 38] yet delivered sequentially as per institutional experience (i.e. first delivery up to 60 Gy + second / re-irradiation delivery up to 40 Gy with at least 6–12 months in between treatments); in the case of re-irradiation exceeding a timeline of 12 months, a 5% annual dose decay from the total dose was also applied when necessary (as per institutional experience).

**Variables defining PPD-adaptation for GKRS 2 and GKRS 3:**

- The lesion was re-planned on the LGP system in accordance with volume dynamics picked up on the ensuing stereotactic MRI, corresponding with radiological signs of early response, defined as volume reduction by > 1cm3 on contrast-enhanced 3D T1 FSPGR and/or evidence of intratumoral necrotic changes (non-enhancing areas with high T2 signal) as per institutional guidelines and literature-based recommendations [39]. A new PPD was generated by adding 0.5–1 Gy (approximate interfraction BED increment of 5–10 Gy) to the previous prescription (GKRS 2 and 3) with the aim to adaptively optimise cell kill from the margin inwards [40]. More information on the rationale behind this step is found in the discussion.
- To achieve steep escalating dose distributions inside the lesion while concurrently increasing the second prescription described below (V_10Gy_), lower prescription isodose lines (35–40%) along with a higher number of isocentres were regularly applied [41].

#### Second prescription: intratumoral 10 Gy-volume modulation (V_10 Gy_ in %) to trigger ablation

The second prescription aimed to circumscribe the possible uncertainties generated by BED calculations in relation to (i) intratumoral dose distributions >8–10 Gy/fraction and (ii) α/β ratio shifts between histological subgroups [31]. Based on previous institutional experience and previous studies on the radiobiology of hypofractionation such as reoxygenation, reperfusion and vascular damage [12, 21, 26, 28, 42], the second prescription was structured on a baseline ablative isodose line of 10 Gy. The aim was to take advantage of key biological dose-dependent events while modulating steep intratumoral dose escalation inside the V_10Gy_ (dose dissipation beyond the 10 Gy-isodose line) to boost ablation at each fraction [4–7].

**Variables defining the intratumoral V**_10Gy_ **at GKRS 1–3:**

- For GKRS 1, we aimed to use the maximal intratumoral 10 Gy coverage (V_10Gy_ in %) achievable without compromising the PPD. Within this framework, a distinction was made between sensitive and radioresistant lesions, where a value of at least 70% and 80% coverage was to be achieved, respectively (when possible).
- For GKRS 2–3, based on volumetric data obtained from the LGP system at each MRI, the V_10Gy_ was re-planned at each stage and increased by 5–10% compared to GKRS 1 when feasible, provided a tumoural reduction by > 1cm3 on contrast enhanced 3D T1 FSPGR and/or evidence of intratumoral necrotic changes (non-enhancing areas with high T2 signal) as previously described [39]. Hot spots were tailored to the solid parts of the lesion when possible.
- Although not a prescription volume, the V_5Gy_ was used to monitor dose dissipation outside the target (treatment quality assurance), aiming to keep it to a constant, or if possible, decrease it throughout treatment (GKRS 1–3).

Illustrative examples on brainstem and non-brainstem location can be found in the list of appendices (Appendix 1 and 2).

### Follow-up

**Fig. 1:**
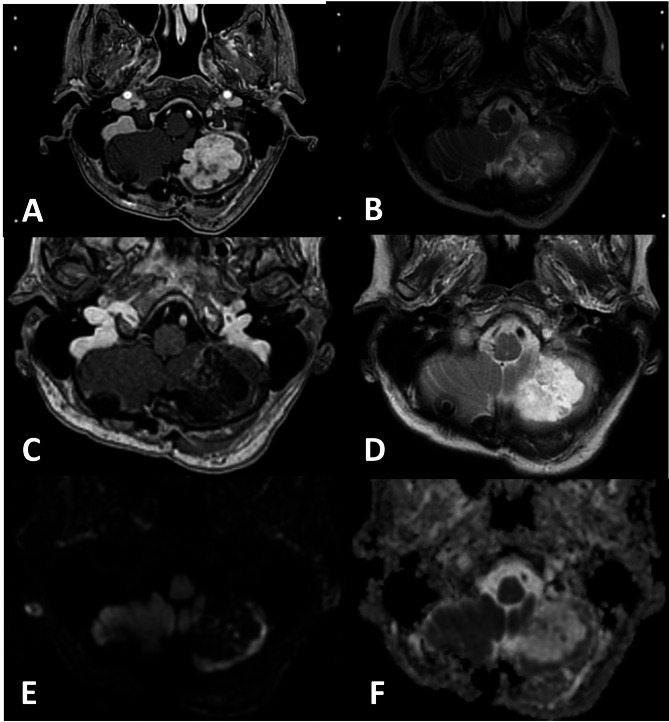
Examples of Adverse Radiation Events (ARE). A and B, stereotactic planning MRI corresponding to GKRS 1 demonstrates a lobulated, intensely enhancing, untreated viable metastasis in the left cerebellar hemisphere (**a**) with heterogeneous low signal on T2 (**b**). C to F: Follow-up MRI 13-months following RRR demonstrates no lesion size progression, with predominant markedly low signal on contrast enhanced T1 with mild streaky partial enhancement (**c**) and high signal on T2 (**d**), with no restricted diffusion evidenced by low signal on Diffusion Weighted Imaging (DWI) (**e**) and no regions lower than normal brain on the Apparent Diffusion Coefficient (ADC) image (**f**) consistent with radiation necrosis / ARE

Where possible, patients were radiologically reviewed at 1, 2, and 4 months post RRR, and then every third or fourth month, for a minimum of 5 years. For validation purposes, the radiologic data for all patients were retrospectively evaluated by experienced Gamma-Knife surgeons, neuroradiologists and qualified medical physicists. Early and late ARE were identified (an example shown in Fig. 1) and monitored based on symptomatology and serial imaging assessment, which included RANO-BM guidelines and literature-based recommendations [39, 43, 44], and are summarised as follows:

1. Potential increase of tumour volume with central necrosis (non-enhancing areas with high T2 signal, alongside local contrast enhancement on T1-weighted MRI).
2. Lack of progression of tumour volume on serial imaging.
3. Possible T2-signal changes (hypointense signal in the solid region indicating viable tumour, whilst hyperintense T2 signal in the areas of ARE/necrosis).
4. Patterns of restriction on diffusion-weighted MRI (absence of restriction was indicative of ARE, while evidence of restriction suggested a viable tumour).
5. Increased dynamics on DSC T2* perfusion-MRI (markedly increased relative Cerebral Blood Volume [rCBV] compared to white matter reference region, usually indicating viable tumour, while no increase in rCBV suggested ARE)
6. Metabolic uptake of complementary FDG or methionine-PET indicated a viable tumour when MRI examinations were deemed inconclusive [44].

From a statistical standpoint, Cox regression analysis was used to identify independent predictive factors of survival and toxicity, including the following variables: age, total target-volume, brain radiation prior to GKRS 1, KPS at GKRS 1, RPA at GKRS 1, systemic therapy prior to GKRS 1 (appendix 3). Kaplan-Meier (KM) curves plotting overall survival (OS) and LTC were to be used to reenforce survival analysis (appendix 4).

## Results

### Whole cohort

Frame application was well tolerated by all patients. The median overall survival was 7.3 months. Survival at 6, 12, 24, 36, 48, and 60 months were 62%, 41%, 17%, 14%, 10%, and 7%, respectively, in keeping with the KM curve for OS (appendix 4). Due to the limited number of patients, only Cox regression analysis for single-variable relations was deemed reliable enough to study predictors of survival for the entire cohort; in that respect, only age, best KPS and RPA had a *p*-value < 0.05, demonstrating a statistically significant impact on OS (appendix 3). The complementary hazard ratios (HR) breakdown showed that higher age and RPA-class were related to worse survival, while higher KPS was associated with the highest survival (appendix 3). Again, due to the restricted number of patients, the KM curve for LTC proved unachievable. Further subgroup analysis plotting histology vs. LTC was also heavily precluded due to the small number of patients with verified recurrence (*n* = 4; 1 melanoma, 2 breast, 1 lung); however, in accordance to our observations, breast histology displayed best survival trend (8–71 months post treatment), computing 7 cases in the whole cohort, of which 5 survived at least 1 year; lung histology accounted for poorer trends (1–15 month across the entire cohort), with only 2 cases surviving 12 months at least. We saw no obvious survival trend associated to radioresistant histology. The cumulative PPD per fraction for the whole cohort ranged between 6.0 and 8.5 Gy, 6.0–9.0 Gy and 7.0–9.0 Gy for GKRS 1–3, respectively. For radiosensitive histology, the average PPD was 7.4 Gy, 7.9 Gy, and 8.2 Gy for the first, second and third fraction, respectively. For the radioresistant subgroup, the average PPD was 8.2 Gy, 8.5 Gy, and 8.8 Gy for the first, second and third fraction, respectively. In the same fashion, for the radiosensitive subgroup, the intratumoral V_10Gy_ was 69%, 78% and 84% for GKRS 1–3. For radioresistant histology, the V_10Gy_ covered 84%, 88% and 92% of the tumour bed for each consecutive fraction. All 29 patients completed treatment (Tables 1 and 2).

**Table 1:** General data for patients surviving less than 12 months

#	Primary cancer	KPS - RPA	BM location	BM volume at GKRS 1 (cm^3^)	Previous RT with impact on target	Estimated regional tolerance (Gy)(EQD2 / 3 fractions)	First prescription: dose at the margin per fraction (Gy)(GKRS 1/2/3)	Second prescription: V_10Gy_ coverage per fraction (%) (GKRS 1/2/3)	Survival (months)
1	Breast	100–2	Brainstem	3.7	Near target Linac-based RT (6 Gy x 5 + boost 8 Gy x 5) 17m prior.	Max 50 Gy/ 18-21	6.0/6.5/7.0	44/69/75	11
2	Ovary	70–3	Brainstem	4.8	-	54-60 / 21-24	7.0/7.0/8.0	59/69/78	8
3	Breast	70–2	Cerebellum	13.8	WBRT ca 3 years prior.	70-74*/ 21-27*	6.0/6.5/7.0*	53/60/68	7
						*WBRT - 100 Gy local tolerance + annual decay	*Inferior dose prescription due to frailer KPS and sensitive histology		
4	Mesothelioma	70–3	Brainstem (extrinsic)	10.2	-	54-60 /21-24	8.0/8.5/9.0	79/90/94	7
5	Lung	70–3	Parieto-occipital region	29.8	-	54-60 /21-24	8.0/9.0/9.0	76/92/93	6
6	Colorectal	90–2	Occipital lobe	50.1	-	60 / 24	8.5/9.0/9.0	90/94/97	6
7	Colorectal	70–2	Cerebellum met 1	12.9	-	Up to 100 Gy tolerance (each lesion) / 24-27	8.5/9.0/9.0	89/93/96	4
			Cerebellum met 2	3.6			8.5/9.0/9.0	88/92/99	
8	Melanoma	70–2	Cerebellum	15.1	-	Up to 100 Gy tolerance (each lesion) / 24-27	8.5/8.5/9.0	82/83/94	3
9	Colorectal	70–2	Fronto-parietal region	24	-	54-60 / 21-24	8.0/8.5/8.5	83/90/90	3
10	Lung	90–2	Brainstem	1.8	-	54-60 / 21-24	8.0/8.5/8.5	86/92/92	2
11	Mesothelioma	60–3	Parietal lobe	25.1	-	54-60 / 21-24	8.0/8.0/8.5	80/81/83	2
12	Lung	60–3	Thalamus	9.5	-	50-54 / 21	7.5/8.0/8.0	72/81/80	1
13	Lung	90–2	Temporal lobe	10.2	-	54-60 /21-24	7.5/8.0/8.5	72/81/90	1
14	Lung	70–2	Brainstem	7.1	-	54-60 / 21-24	8.0/8.0/8.0	75/78/75	1
15	Colorectal	80–2	Cerebellum met 1	22.2	-	Up to 100 Gy tolerance / 24-27	7.5/8.0/8.0	73/81/83	1
			Temporal lobe met 2	9.4		54 Gy/21	7.5/8.0/8.0	77/84/81	
16	Lung	70–2	Thalamus	3.9	-	50-54 / 21	7.0/7.5/8.0	73/75/88	1
17	Lung	90–2	Brainstem	3	-	54-60 / 21-24	8.0/8.0/8.5	69/74/80	1

BM = Brain metastasis, GKRS = Gamma Knife radiosurgery, RT = Radiotherapy, Linac RT = linear accelerator radiotherapy, EQD2 = Equivalent dose in 2Gy fractions, KPS =  Karnofsky Performance Status (Score), RPA = Recursive Partitioning Analysis (Class), m = months

**Table 2:** General data for patients surviving 12 months or more

#	Primary cancer	KPS - RPA	BM location	BM volume at GKRS 1 (cm^3^)	Previous RT with impact on target	Estimated regional tolerance (Gy) (EQD2/3 fractions)	First prescription: Dose at the margin per fraction (Gy) (GKRS1/2/3)	Second prescription: V_10Gy_ coverage per fraction (%) (GKRS1/2/3)	Survival (months)
1	Breast	90–2	Brainstem	5.2	-	54-60 / 21-24	7.5/7.5/8.0	69/70/83	117 (alive)
2	Thymus	100–1	First RRR: Temporal + parietal lobe (2 mets)	25 +14.5	Linac-based RT 4 m prior (6 Gy x 5 close to targets)	54-60 / 21-24	8.0/8.5/8.5	79/85/83	115 (alive)
						54-60 / 21-24	8.0/8.5/9.0	82/81/90	
			Second RRR: Temporal lobe (local recurrence post linac based RT)	2.1	Linac-based RT 18m prior (same as above) + first RRR close to target 14m prior	40-50* / 18-21*	7.5/8.0/8.0	85/87/88	
						*Overlapping with previous RRR and LINAC treatment			
3	Breast	80–2	Brainstem	0.3	WBRT ca 15 m prior	24-30 / 15-18	7.0/8.0/8.0	48/77/74	61
				0.5			7.0/8.0/8.5*	49/68/83	
							*Higher dose due to small volume, acceptable KPS, and need for LTC)		
4	Melanoma	100–2	Brainstem	1.4	-	54-60 / 21-24	8.0/8.0/8.5	78/79/86	40
5	Breast	100–1	Frontal lobe (Motor cortex)	7.4	-	54-60* / 21-24*	8.0/9.0/9.0	80/95/96	32
						*Motor fibres avoided using TMS pre-GKRS			
6	Colorectal	100–2	Cerebellum	9.2	WBRT 11m prior	70*/ 24-27*	8.5/9.0/9.5	92/97/99	23
						*WBRT - 100 Gy local tolerance + annual decay			
7	Renal	90–2	Parietal lobe	12	-	54-60 / 21-24	8.5/8.5/9.5	84/87/95	21
			Frontal lobe	17.3		54-60 / 21-24	8.5/8.5/9.0	89/89/96	
8	Caecum	90–2	Cerebellum	17.3	-	Up to 100 Gy tolerance / 24	8.0/8.5/9.0	85/92/95	17
9	Breast	80–2	Brainstem	2.9	-	54-60 /21-24	8.0/8.5/9.0	82/83/94	16
10	Lung	90–2	Brainstem	9.2	WBRT 7m prior	20-30 /15-18	6.0/6.0/7.0*	43/47/64	15
							*Higher dose not given due to large lesional volume		
11	Breast	70–2	Cerebellum	18	WBRT 10m prior	70*/ 21-27*	7.5/8.0/8.5	79/89/95	13
						*WBRT - 100 Gy local tolerance + annual decay			
12	Lung	80–2	Temporal lobe	9.2	-	54-60 / 21-24	7.5/8.0/8.5	77/77/89	12

BM = Brain metastasis, GKRS = Gamma Knife radiosurgery, RT = Radiotherapy, Linac RT = linear accelerator radiotherapy, EQD2 = Equivalent dose in 2Gy fractions, KPS =  Karnofsky Performance Status (Score), RPA = Recursive Partitioning Analysis (Class), m = months

### Sub-cohort surviving less than one year (*n* = 17)

Patients surviving less than one year (*n* = 17) died of general disease progression and not from the treated lesions. No symptomatic ARE were accounted for. 11 patients survived < 6 months (1–4 months), of which 5 patients died prior to their first follow-up MRI, yet without neurological decline. 6 patients survived at least 6 months (6–11 months). In those able to undergo MRI surveillance (*n* = 11), the rate of LTC was 100% at last follow-up. 2 patients had radiotherapy prior to adaptive treatment and 3 had WBRT post-adaptive treatment due to ‘out-of-field’ disease. The RPA status for all patients surviving less than one year was at least 2 with a KPS precluded to 60–70 in 60% of the cohort (*n* = 11). 41% of the sub-cohort (*n* = 7) had radioresistant histology, including 2 cases of mesothelioma, 1 case of melanoma, and 1 case of renal cancer. Of note, out of those surviving 6 months or less (*n* = 13), 6 had radioresistant lesions. The cumulative PPD ranged between 6.0 and 8.5 Gy, 6.5–9.0 Gy and 7.0–9.0 Gy for the 1st, 2nd and 3rd fraction, respectively. The intratumoral V_10Gy_ ranged between 44 and 90% for the first fraction, 60–94% for the second fraction and 75–99% for the last fraction. More information can be found on Table 1.

### Sub-cohort surviving at least one year (*n* = 12)

#### Clinical and radiological characteristics

The sub-cohort of patients surviving at least 1 year (*n* = 12, 41% of the whole cohort), consisted of seven females and five males with a median age of 55 years (Table 2). Tumour histology in descending order included breast (*n* = 5), lung (*n* = 2), colorectal (*n* = 2), malignant melanoma (*n* = 1), renal (*n* = 1) and thymus (*n* = 1). KPS was 70–100 with a median of 90, while RPA ranged between 1 and 2. Five patients had received radiotherapy 6–12 months prior to adaptive treatment (WBRT *n* = 4, LINAC-based hypofractionated radiation *n* = 1). The initial tumour volume ranged between 0.3 cm^3^ and 25 cm^3^ with an average of 8.9 cm^3^. Four of the twelve patients had visible oedema on T2-weighted images acquired on the treatment day, their volume ranging between 13 cm^3^ and 121 cm^3^.

#### Dosimetric values

The cumulative dose per fraction ranged between 6.0 and 8.5 Gy, 6.0–9.0 Gy and 7.0–9.5 Gy for 1st, 2nd, and 3rd fractions, respectively (Table 2). The average prescribed dose to radiosensitive histology (lung, breast, ovarian, and thymus) was 7.5 Gy, 8.0 Gy and 8.4 Gy at each fraction, respectively. For radioresistant histology (renal cell carcinomas, malignant melanoma, colorectal adenocarcinomas), the prescribed doses were 8.3 Gy, 8.5 Gy and 9.1 Gy at each fraction, respectively. As for the second ablative prescription, the average tumour bed coverage by V_10Gy_ study guidelines was 70%, 78% and 85% for radiosensitive histology whereas 85%, 89% and 94% for radioresistant lesions.

#### Survival data

**Fig. 2:**
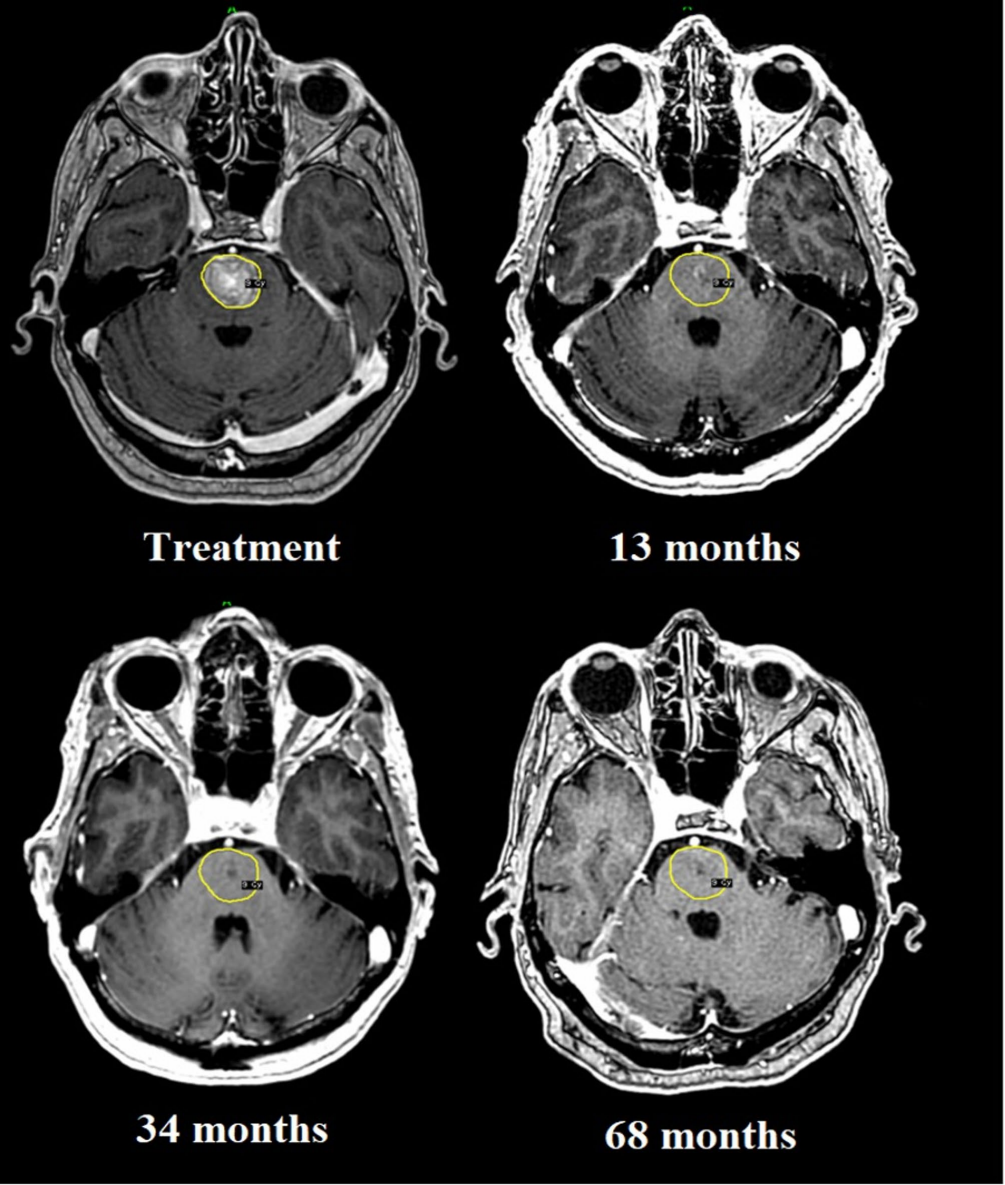
Intrinsic brainstem metastasis treated with RRR (breast cancer histology), corresponding to survivor 1; target volume 5,2 cc at GKRS 1, hardly measurable 68 months post RRR. The patient was still alive at the time of paper submission (117 months post-RRR)

**Fig. 3:**
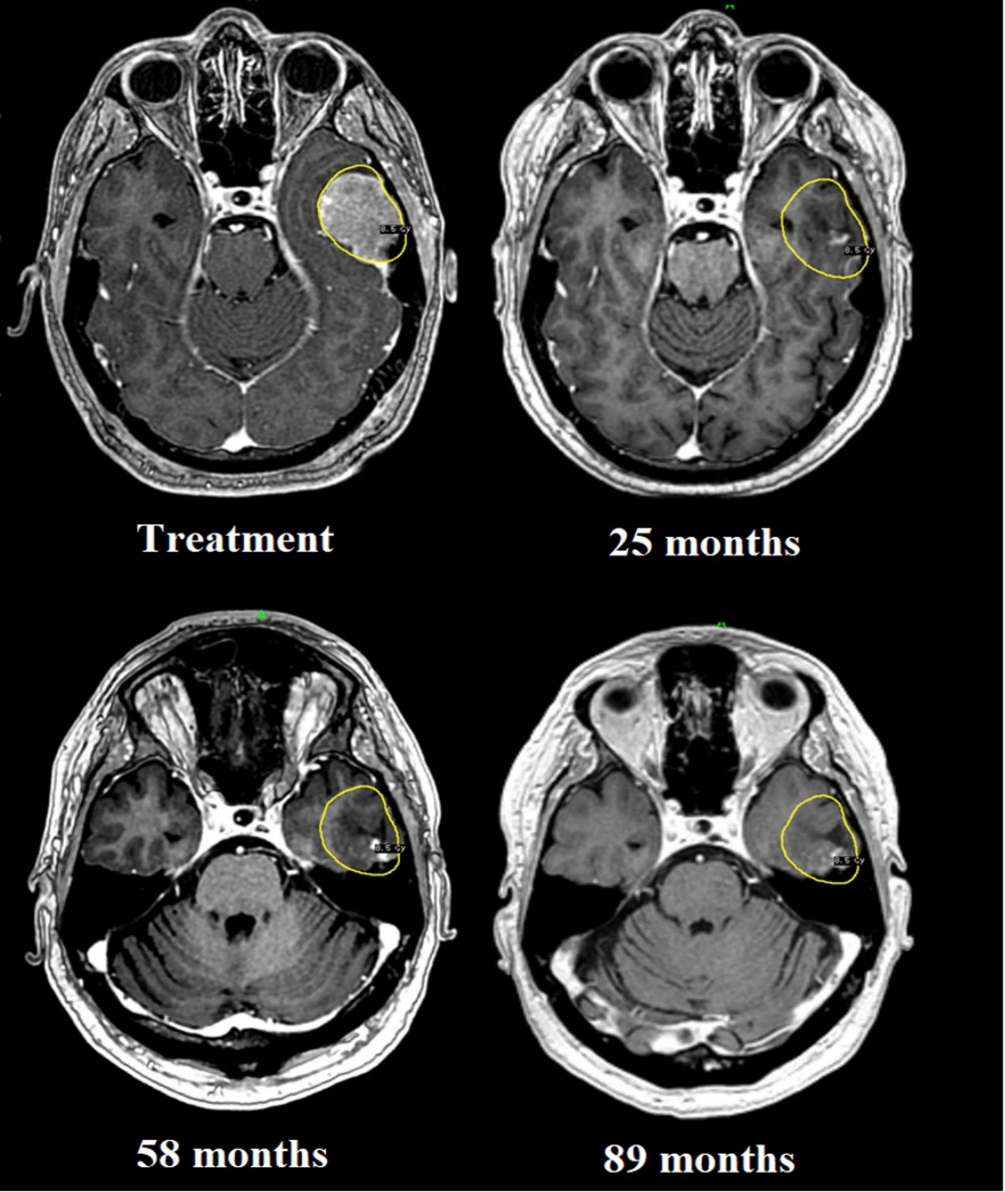
Left temporal metastasis in a patient with thymic cancer (Survivor 2); the images show almost complete regression of the temporal lesion 25 months post RRR, with sustained local control 89 months post treatment. The patient was still alive at the time of paper submission (115 months post-RRR)

The LTC rate at one year for this sub-cohort was 75% (*n* = 9). In those patients surviving at least 24 and 36 months, the LTC rate remained stable (80% and 75%, respectively). 4 cases (patients 4, 9, 11, and 12) developed MR- and/or PET-verified local recurrences at intervals ranging between 6 and 20 months. The corresponding histology for these four cases accounted a malignant melanoma metastasis in the brainstem, a breast cancer metastasis in the brainstem, a breast cancer metastasis in the cerebellum and a lung cancer metastasis in the temporal lobe. The first patient underwent salvage surgery but died shortly after of general disease progression. Neither of the remaining three received any treatment post recurrence. Despite proven focal relapse, patients 4, 9, 11, and 12 survived 40, 16, 13 and 12 months post-adaptive treatment, respectively. All of these patients succumbed to general disease and not the treated target. Finally, at the time of paper submission, 2 patients were still alive (115- and 117-months years post-adaptive treatment, respectively), with stable extra-/intracranial disease status while retaining a KPS of 100. Treatment and post-treatment images of these 2 patients are shown in Figs. 2 and 3, respectively.

#### ARE features

Four patients had MR-verified asymptomatic ARE, ranging between 6 and 20 months (patients 3, 6, 8 and 10). One case (patient 2) developed symptomatic ARE approximately 5 months post-adaptive treatment, and responded well to steroids and anti-epileptic medication; this patient is still alive at the time of submission with no major neurological deficit (115 months post adaptive radiosurgery).

## Discussion

### Long-term outcome: interpretation of collected data

With best supportive care being the only alternative, the outcome of adaptive GKRS delivered in subacute accelerated settings (with a 72-hour interval) was promising. Firstly, 41% of the initial 29 patients (*n* = 12) survived at least one year. Secondly, those surviving less than one year (*n* = 17) succumbed to general disease progression with no further decline of neurological function, with proven local control in those able to undergo follow-up imaging. Previous studies have associated large intracranial tumour volume and frail performance with shorter survival [14, 15, 21, 45–49]. In this study, however, only age, KPS and RPA were seen as significant predictors of survival following Cox regression analysis (appendix 3). In addition, the average KPS and RPA-class for those surviving more than one year was 90 and 1–2, respectively, while those who died prior to one year follow-up were 70 and 2–3, respectively. Of note, even if tumour volume was not correlated to survival by Cox regression analysis (appendix 3), in the subgroup of patients surviving less than 12 months, tumour volume estimates exceeded those surviving one year or more by almost 2-fold (total volume 260.2 cm3 vs. 134.2 cm3). As such, lesional volume may still have played a role in the outcome of treatment. Sadly, the limited number of patients precludes any statistical corroboration in that respect. Adding to the complexity, the inclusion of both radiosensitive (e.g., breast, lung, thymus, ovarian) and radioresistant (e.g., melanoma, renal, mesothelioma, colorectal) tumours in this small cohort may have confounded LTC rates, difficult to overcome for the same numerical restrictions; however, although no reliable analytical data in relation to response and survival by histology subgroup could be obtained, best survival trends were noticed in the breast subgroup (8–71 months post treatment), accounting for 7 cases in the whole cohort, of which 5 survived at least 1 year. These results can be explained by the histology itself, although in a confounding manner, best KPS/RPA-class status may have also played a substantial role, as demonstrated by single-variable cox-regression analysis (Appendix 3). In contrast, worse survival was seen in lung histology (1–15 months across the entire cohort), with only 2 cases surviving 1 year or more, possibly due to substandard KPS/RPA class status at the time of inclusion. Captivatingly, although almost 40% of all cases presented with radioresistant histology, widely associated with recurrence and therefore a precluded survival [50, 51], we saw no such clear trend across the whole cohort. This raises the question as to whether a modulated dose increment per fraction (PPD- and V_10Gy_) may have contributed to this positive evolution. Of interest, 24% of all cases had undergone previous brain radiotherapy (7 out of 29 patients). Although previous brain radiation had no impact on survival on Cox regression, almost all patients in this subgroup (5 out of 7 patients) survived 12 months or more, which could make a case for the protocol used here. Indeed, despite a dismal prognosis limited to weeks prior to starting treatment, 18 out of 29 patients survived at least 6 months (62% of the total population) with no further decline of the local neurological function, hence avoiding neurological death (either by direct response, delayed growth, or absence of life-threatening ARE).

Exploring the literature for recent standard hypofractionated (Table 3) and staged procedures (Table 4), we identified comparable rates of survival despite the shorter timeline of our procedure. Executing a re-analysis of three-staged GKRS for metastatic brain lesions larger than 10 cm^3^ (two separate studies comprehending 78 and 43 patients, respectively), Yamamoto et al. reported similar survival times to those from our study, with concurring neurologic death and decline incidences set at about 14 and 18%, respectively [72] (Table 4). On the other hand, the rates of LTC for the whole cohort at one-year in the context of our research work, did not match those listed on Tables 3 and 4, potentially explained by some of the adverse factors intrinsic to this study, such as heavily precluded survival, limited number of patients, and heterogeneity of histology. Nonetheless, it remains important to highlight that in the sub-cohort surviving at least 12 months, the rate of LTC at one year was compatible with the majority of studies included in our review (75%, i.e. 9 out 12 patients), with a trend on remaining constant on those surviving at least 24 and 36 months (Tables 3 and 4).

**Table 3:** Literature review of studies on hypofractionated stereotactic radiotherapy (HFSRT)

Author	Title	Study Type	Technique Used and treatment schedule	Median Dose	Number of Patients	BM	Median Follow-up (months)	Local Control Rate at 12 months	OS at 12 Months	PFS at 12 months	Extra data
[52]	Hypofractionated Stereotactic Radiation Therapy for Intact Brain Metastases in 5 Daily Fractions: Effect of Dose on Treatment Response	Prospective (presumably as per description)	HFSRT	30 Gy in 5F (Median Total)	220	334	10.8	76.2%	OS 11.8 months	35.1%	OS 48.2% at 24 months.
[53]	Brain metastases: Single-dose radiosurgery versus hypofractionated stereotactic radiotherapy	Retrospective	SRS (a) vs. HFSRT (b)	(a) 16 Gy single fraction (b) 30 Gy in 5-6F(Median Total)	97	135	10.0	67% (all patients)	62% (a) vs 70% (b)	60% (a) vs 69% (b)	Chronic toxicity more frequent in the HFSRT group.
[54]	Stereotactic irradiation of non‑small cell lung cancer brain metastases (Cyberknife™-based SRS and SRT): evaluation of local and cerebral control in a large series	Retrospective	SRS (a) vs. HFSRT (b)	(a) 20-25 Gy (b) 24-36 Gy in 3-5F (b)	100	At least 143	33.0	78.7%	44%	See ‘extra data’	Cumulative incidence of local progression at 1 year 21.3% (RECIST 1.1) vs 21.9% (RANO-BM)
[55]	Brain metastases treated with hypofractionated stereotactic radiotherapy: 8 years’ experience after Cyberknife™ installation	Retrospective	Surgery and HFSRT (a) vs. HFSRT alone (b)	Three different schedules compared: 27 Gy in 3F, 30 Gy in 5F, 35 Gy in 5F	389	400	40.0	87.02% (a) vs. 73.53% (b)	61.43% (a) vs. 50.13% (b)	N/A	Late toxicity (radionecrosis) limited to 5%
[56]	A multi-centre analysis of single fraction versus hypofractionated stereotactic radiosurgery for the treatment of brain metastasis	Retrospective	SRS (a) vs. HFSRT (b)	(a)- <2cm, 20-24 Gy, 2-3cm, 18 Gy, 3-4cm, 15 Gy (b) 15-30 Gy in 3 F, 25-30 Gy in 5 F, 16 Gy in 2 F)	156	335	12.0	91% (a) vs. 85% (b)	46% (Distant brain failure for entire cohort)	N/A	ARE incidence at 1 year: 10% (a) vs 7% (b)
[57]	Fractionated Stereotactic Radiation Therapy Using Volumetric Modulated Arc Therapy in Patients with Solitary Brain Metastases	Retrospective	HFSRT	25-40 Gy in 5F	46	46	11.0	86%	N/A	N/A	ARE-rate (grade 3 or worse): 11%
[58]	Retrospective analysis of hypofractionated stereotactic radiotherapy for tumours larger than 2 cm	Retrospective	HFSRT (BM <3cm (a), BM >3cm (b))	35 Gy in 5F	45	58	11.3	67.1% (a) vs 61.5% (b)	15.1 months (a) vs 13.7 months (b)	54.6% (a) vs 36.9% (b) (brain progression free survival at 1 year)	Only 2 cases of late toxicity (5-6 years post-HFRST)
[59]	Radiosurgery or hypofractionated stereotactic radiotherapy for brain metastases from radioresistant primaries (melanoma and renal cancer)	Retrospective	HFSRT (a) vs. SRS (b)	(a) 27-33 Gy in 3F, 30-36 Gy in 6F (range) (b) 18-25 Gy single F	60	193	7.4	74% (LPFS for the entire cohort)	45% (Entire cohort)	72% (a) vs. 79% (b) [LPFS per group]	ARE-rate 7.1% (a) vs. 9.6% (b).
[60]	Clinical Outcomes of Patients with Limited Brain Metastases Treated with Hypofractionated (5 × 6 Gy) Conformal Radiotherapy	Retrospective	HFRT (conformal)	30 Gy in 5F	195	231	12.8	83%	53%	N/A	N/A
[61]	Fractionated stereotactic radiation therapy forintact brain metastases	Retrospective	HFSRT	25-30 Gy in 5F	72	182	5.0	Entire cohort: 86% By Size: 95% and 61% (<3cm and >3cm). By dose: 91% treated with 30Gy vs 75% with 25Gy.)	29%	N/A	ARE-rate 6% and 33% at 6 and 12 months.
[62]	Single dose versus fractionated stereotactic radiotherapy for brain metastasis	Retrospective	SRS (a) vs HFSRT (b)	(a) 20 Gy (b) 36 Gy in 6F	98 (58 SRS, 40 HFSRT)	109	7.0	71% (a) vs 69% (b) [LPFS]	36% (a) vs 31% (b)	43% (a) vs 65% (b) [RPFS]	ARE-rate 17% (a) vs 55(b). Neurologic mortality 17% at 1y, 44% at 2y.

N/A (Not Available) refers to (i) information not found in the main text, (ii) data solely shown on figures/diagrams and (iii) non-relevant/unavailable additional feedback. BM = Brain metastases, SRS = Stereotactic Radiosurgery, GKRS = Gamma Knife Radiosurgery, OS = Overall Survival, LPFS = Local progression free survival, RPFS=Regional progression free survival, ARE = Adverse Radiation Effect

**Table 4:** Literature review of staged procedures

Author	Title	Study Type	Technique used and treatment schedule	Dose prescription	Number of Patients	Number of BM	Median Follow-up (months)	Local Control Rate at 12 months	OS at 12 Months	PFS at 12 months	Extra data
[63]	Two-staged stereotactic radiosurgery for the treatment of large brain metastases: Single institution experience and review of literature	Retrospective	2 staged GKRS, circa 4 weeks interval	12-15 Gy per session (range).	12	23	6.4	N/A	N/A	N/A	4 BM (3 pats) relapsing 106-157 days post treatment
[64]	Adaptive Staged-Dose Gamma Knife Radiosurgery for the Treatment of Large Brain Metastases: Report of 40 Consecutive Cases and Analysis of Literature	Retrospective	2 staged GKRS, 4 weeks interval	12-14 Gy Gy per session (range)	40	30	6.0	N/A	N/A	N/A	75% survival rate at 3 and 6 months. Local control at 3 and 6 months, 100% and 96.7%.
[65]	Management of multiple brain metastases by staged SRS focusing on utmost risk lesions (abstract available only)	Retrospective	2 and 3 staged SRS	20-24 Gy within 2 staged GKRS; 24 Gy within 3 sessions	30	N/A	N/A	N/A	N/A	N/A	Remission rate of utmost risk and less critical lesions: 100% and 95.16%. ARE grade 3 in 1 patient.
[66]	Staged Stereotactic Radiosurgery as a Novel Adaptive Approach to Salvage Previously Irradiated Brain Metastases (abstract available only)	Retrospective	3-staged SRS, median interval 2.6 weeks	4-8 Gy per session (range)	24	55	9.0	N/A	N/A	N/A	Local failure rate 27% (median time 3.4 months). Asymptomatic ARE observed in 4/55 pats (7%).
[67]	Staged Stereotactic Radiosurgery for Large Brain Metastases: Local Control and Clinical Outcomes of a One-Two Punch Technique	Retrospective	2-staged GKRS, 1 month interval	10-21 Gy per session (range)	33	39	7.7	86.7% (assumed)	60%	N/A	Local failure at 6 and 12 m: 3.2 and 13.3%. ARE reported in 4/39 pats. Neurologic death associated to GKRS in 2 cases.
[68]	Three-stage Gamma Knife treatment for metastatic brain tumors larger than 10 cm3: a 2-institute study including re-analyses of earlier results using competing risk analysis	Retrospective	3-stage GKRS, M (a) vs C series (b)	10 Gy per session with 2 weeks interval (Small lesions treated with 19-24 Gy single fraction)	78 (a) vs 43 (b)	N/A	16.0	89.6% (a) vs 85.6% (b)	35.2% (a) vs 26.4% (b)	N/A	Median OS: 8.3 for (a) and 8.6 months for (b). Survival rates at 6, 24, 36 months for (a) 55.2%, 22.1%, 7.7%. For (b) 62.5%, 7.2% 2.4%
[69]	Impact of 2-staged stereotactic radiosurgery for treatment of brain metastases ≥ 2 cm	Retrospective	2-staged SRS	12-18 Gy per session (range), median interval 34 days.	54(Favourable group=35 vs unfavourable= 19)	63	N/A	88% at 6 months (estimated equivalence from 12% TTP at 6 months).	Median OS 10.8 months (Median OS for the favourable group 26.0 months vs 3.0 for the unfavourable group).	N/A	7 cases of ARE (4 symptomatic).
[70]	Multisession gamma knife surgery for large brain metastases	Retrospective	Multisession GKRS	(i) Three session GKRS, 10 Gy per session with 2-week interval. (ii) Two session GKRS, 10-13 Gy per session with 1–4-week interval.	56	65	6.0	N/A (read PFS column)	42%	80%	Median survival 7 months (6- and 18-month survival rate: 62% and 31%). Mean PFS 29.0 months (6- and 18-month PFS rate: 93% and 74%).
[71]	Three-staged stereotactic radiotherapy without whole brain irradiation for large metastatic brain tumours	Prospective	3-staged SRS	10 Gy per session with 2-week interval	43	46	7.8	75.9%	N/A	80.7%	Median OS 8.8 months. Neurological and qualitative survivals at 12 months: 81.8% and 76.2%.

N/A (Not Available) refers to (i) information not found in the main text, (ii) data solely shown on figures/diagrams and (iii) non-relevant/unclear/unavailable additional feedback. BM = Brain metastases, SRS = Stereotactic Radiosurgery, GKRS = Gamma Knife Radiosurgery, OS = Overall Survival, PFS = Progression free survival, TTP = Time to progression, ARE = Adverse Radiation Effect

Furthermore, although four patients developed local recurrence ranging between 6 and 20 months post-adaptive accelerated staged GKRS (RRR), all of which survived at least one year, with one case succumbing 40 months post-treatment due to a combination of local recurrence and generalised disease. Two of these four relapsing patients had negative adjunct factors to treatment outcome (both were of brainstem location, with one having radioresistant histology) [50, 51, 73]. In contrast, in the same sub-group surviving at least one year, 7 out of 12 patients (58% of the sub-cohort) showed continuous tumour volume regression from the time of treatment up to the last follow-up MRI, with no neurological complications. This can be partially explained by the radiosensitive character of most of the treated histology and the best KPS/RPA class status at GKRS 1. Again, in the cohort of patients surviving at least one year, extracranial disease activity was the cause of death (and not intracranial recurrence) in all cases, suggesting that the procedure may have played a key role in delaying tumour growth even when local control was not fully achieved. Although 5 patients developed ARE, only one became symptomatic (further explained in section ii). Of note, 4 of 5 patients with ARE had received radiation treatment prior to adaptive accelerated staged GKRS (WBRT, *n* = 3; LINAC-based stereotactic hypofractionated treatment, *n* = 1), explaining ARE development. Moreover, only the patient who underwent stereotactic treatment developed symptoms (the same patient mentioned above and in section ii). This is further explained by BED delivery inevitably exceeding radiotolerance thresholds in the overlapping treatment fields [7]. Another point worth mentioning is the fact that perilesional oedema was present in a third of the whole study population (*n* = 10 of 29) prior to starting treatment, despite ongoing steroid treatment. In the group surviving at least one year, the oedema markedly diminished in all patients from the first GKRS session to the first follow-up scan (e.g. 1-month post-treatment), further confirming the role of stereotactic radiation in the management of tumour oedema, particularly on those poorly responsive to steroids.

### Surviving cases up to date: a closer analysis

Despite an extremely poor pre-treatment prognosis, two patients were still alive at the time of submission (almost 10 years post-treatment) with no signs of local recurrence on serial imaging or neurological sequelae (Figs. 2 and 3). To illustrate how the procedure can be used in selected cases, it is worth explaining the circumstances leading to treatment and the rationale used for GKRS planning.

#### Survivor 1 (117 months post-accelerated adapted GKRS)

The first patient presented with a central pontine breast cancer lesion measuring 5.2 cm3 (Fig. 2). In volumetric terms, the lesion could have been considered for single-dose GKRS; however, by institutional V_10Gy_ constraint guidelines (a maximum of 3cm^3^ for the radiation naïve brainstem and 1cm^3^ with previous radiation), treatment with 18 Gy in one fraction was heavily precluded, partially due to the convoluted volume of the brainstem (roughly up to 30 cm^3^ in the adult population) [74]. Additionally, in our experience, a dose < 18 Gy carries a high risk of recurrence [74] with ensuing detrimental impact on the KPS, ultimately limiting the scope of oncological management in general. As such, a staged approach aiming to 'mimic' the ablative effects of 18Gy single fraction delivery was deemed preferable. RTOG 90 − 05 guidelines [74, 75], as well as expert panels such as the Thames Valley and Wessex Radiotherapy Operational Delivery Network (UK) and the ESTRO ACROP bring further support to this line of reasoning [76]. Furthermore, it is also in keeping with the results from Vogelbaum et al. and Masucci et al., associating worse local control with doses ranging between 15 and 18 Gy [75, 77, 78]. In contrast, multiple studies in the literature have reported a lesser toxic outcome on smaller (often sub-cm^3^) brainstem lesions. A recent review by Nicosia et al. involving 105 patients with 111 BM treated with SRS and hypofractionated radiosurgery confirmed that a median tumour volume ≤ 0.4cm^3^ and concurrent targeted therapy were associated with longer LTC, with combined treatment remaining a strong independent predictor. Interestingly, neurological death was accounted in 30% of the patients, of which 3% being due to local progression [79]. Kawabe et al. [73] reviewed 200 cases with BM treated with single dose GKRS. The mean and median tumour volumes were 1.3cm^3^ and 0.2cm^3^ with a median peripheral radiation dose of 18.0 Gy (range 12.0–25.0 Gy). Optimal KPS, single lesion, and well-controlled primary were predictive for longer survival, which is seemingly in keeping with the results from Liu et al., and the outcome of this study [73, 77]. Despite a median overall survival of 6 months, the neurological survival was reported to be 90.8% 2 years post-GKRS, which further supports the long-term results of this study and previous reports [5, 7, 73]. This is of the utmost importance considering that the window for re-do radiosurgery in the case of recurrence is usually slim, not seldom leading to WBRT and/or neurological death following supportive care management [73]. Following the application of protocol guidelines for brainstem location (as described in appendix 1), the lesion exhibited continuous volume regression without signs of ARE throughout follow-up (27% reduction in 4 weeks from GKRS 1 to first follow-up, hardly measurable on latest serial scans) [6], well in keeping with the results from other groups, ultimately underscoring the benefits of hypofractionation vs. SRS on critically located BMs, including small volume lesions [73, 74, 79–81].

#### Survivor 2 (115 months post-accelerated adapted GKRS)

For the second patient, conditions were more complex [7]. This patient had a background of thymic carcinoma, developing 3 new metastases (M2-M4) only 4 months after undergoing surgical excision and post-operative focal hypofractionated radiotherapy (30 Gy in 5 fractions) for a solitary left temporal metastasis (M1). In fact, M3 was suspected to be an out-of-field recurrence from M1 [7]. By multidisciplinary team assessment, surgery was deemed not indicated due to multifocal metastatic brain disease, suspected failure to previous surgery and radiation, and a MIB index of 50% on prior collected samples. Further focal LINAC-based radiotherapy was also deemed of risk in relation to M1 and M3 and probably not useful in terms of local control. Single fraction GKRS was associated with a high risk of ARE due to the size of the supratentorial BM as well as potential field overlapping. The only option available was WBRT or adaptive, accelerated staged GKRS to M3 and M4 combined with single dose GKRS to the smallest (M2). Considering the known adverse effects of WBRT concurring with a high risk of local failure on lesions > 10cm^3^ [74], the patient opted for the latter and was treated in accordance with our non-brainstem location protocol. The MRI at 4 weeks post-treatment showed a 26% size reduction of M2 (initial volume of 2.7cm^3^ pre single dose GKRS) while M3 and M4 had reduced 90% from its original volume (25.0 cm^3^ to 2.7 cm^3^ and 14.6 cm^3^ to 1.32 cm^3^, respectively) following adaptive treatment [7]. Of further interest, only M2 developed signs of ARE despite its lesser volume. A year later, the patient experienced a further relapse on M1 (M5 = 2 nodules at the surgical margin), again treated with adaptive GKRS despite a degree of overlapping with the previous field of treatments corresponding to M1 and M3 (local previous radiation in BED at least 120 Gy to normal tissue). Following this adaptive intervention, the patient developed transient symptomatic ARE in the form of epilepsy (MRI-verified), subsequently well managed with steroid treatment and longstanding antiepileptic drug therapy. Considering the behaviour of M2 following standard radiosurgery, we suggest that a more aggressive symptomatic ARE was likely to take place should we have chosen to deliver single-dose GKRS on M3, M4 and M5. More details relevant to this case, including dosimetry data, can be found in the medical literature [5–7].

### Factors inherent to treatment strategy and planning

Stereotactic hypofractionated radiotherapy, along with staged procedures, is known to provide optimal rates of LTC with limited ARE [2, 3, 11, 12, 18, 19, 47, 82, 83] when single-dose radiosurgery is deemed less suitable. However, as illustrated by Tables 3 and 4, ARE may still occur. In this complex framework, interlinked variables such as tumour volume, functional topography, histology, extension of perilesional oedema, co-morbidity, and RPA/KPS are also recognised to be crucial factors in outcome [1, 11–13, 34, 47]. Consequently, and in contrast to our patient cohort, radiosurgical procedures are commonly used in patients with better prognosis, not seldom with a life expectancy of 6 months or more. Moreover, volumetric estimates (including dose-volume histograms) have traditionally been the principal deciding factor in the choice of hypofractionated schedules in BM-management; yet, relevant to treatment decision making and GKRS planning, the volumetric definition of ‘largeness’ warrants a modern review. Indeed, radiosurgeons are often compelled to rely on specific ‘one size fits all’ protocols structured on Quantec data and regional policies, not necessarily adjusted to hostile factors such as previous focal radiation or regional neurofunction [16, 17]; in these cases, dose deviations are often applied empirically, based on individual experience, legal considerations, and personal perspective on LQ-model reliability and subsequent BED-estimations. By clinical experience, we know that standard dose-volume planning parameters such as the use V_10Gy_-V_12Gy_ for single fraction delivery [25] and V_18 − 21Gy_ for three fractions [25] to avoid ARE, are not fully reliable, especially in re-do irradiation settings. Therein our idea to conceive a feasibility study to safely transition from ‘standard’ schedules to more ‘holistic’ customised dose prescriptions.

Different groups have tried to approach the subject by using staged stereotactic procedures in more ‘tailored’ fixtures [78, 84, 85]. In this framework, it is worth highlighting the work of Higushi et al. on staged GKRS, the results sharing similar traits to ours. In a first study (2009), 43 patients with heterogeneous brain metastatic histology (tumour volume > 10 cc) were treated with 30 Gy in 3 sessions with 2-week interval in-between sessions. The rates of local control were 89.8% and 75.9% at 6- and 12-months, respectively, with a neurological survival set at 81.8% at one year [78] (Table 4). Then, through a retrospective multi-institutional study (2018), the same group compared the outcome of 2- (11.8–14.2 Gy) vs. 3-staged (9–11 Gy per fraction) GKRS on 212 patients with large brain lesions. Although statistically not significant, the group noted a longer median survival time in the 3-stage cohort compared to the 2-stage group (15.9 vs. 11.7 months, respectively) [84]; notwithstanding this, there was not much difference in the incidences of tumour progression (21.6% vs 16.7% at 12 months), serious ARE (3.0% vs. 4.0% at 12 months), and neurological death (5.1% vs. 6.0% at 12 months) [78].

In parallel, there is mounting evidence to support the use of hypofractionation on lesions deemed small by standard volumetric parameters [80, 81, 86–88]. Yan et al. identified 60 cases with 133 BM treated with fractionated stereotactic radiotherapy (18–32.5 Gy in 3–5 fractions); the mean tumour size was 1.30 cm (0.57–4.80 cm) and location was a determinant factor in treatment decision making [80]. Local failure at 12 and 24 months was 17.8% and 32.4%, respectively, regardless of tumour size (> 1 cm defined as large and ≤ 1 cm as small); also, irrespective of size, the rate of ARE was 7.1% and 13.2% at 12 and 24 months. In addition, while the rate of LTC at one year for overall lesions was 82.2%, the group with small BM (< 1 cm) showed better control rates (86.8%) under the same period of follow-up [80]. Putz et al. further explored a modern biological rationale to this approach [81]. On a longitudinal volumetric analysis comprehending 120 cases and 190 BM, the group compared the outcome of fractionated radiotherapy (51.6% of cases) vs. SRS (48.4%) on small lesions (mean volume 4.66 cm^3^ vs. 0.40 cm^3^, respectively); nearly congruent with our results, the median time from radiotherapy to death was 10.4 months with a meantime follow-up of 7.4 months. Furthermore, although no significant difference in overall survival was noticed between both groups, the numerical survival was lower in the SRS arm (7.5 months) compared to the fractionated one (11.5 months), despite a preference for SRS on smaller volumes [88]. In addition to protocol-based institutional praxis [76], further studies querying the effects of hypofractionation on lesions < 3 cm have shown similar outcomes over recent years, which bears the hallmark of a new attitude towards BM management, likely to become state-of-the-art in the near future [81, 86–89]. Given the above, we suggest that the definition of ‘largeness’ should not rest on solely volume criteria but on all co-existing factors able to affect the perilesional environment, including prior focal radiation, degree of response to previous radiation (in and outside the CNS), critical location, type of histology, and clinical performance (KPS/RPA-class) [14, 15, 80, 81, 86–88].

In the context of treatment planning strategy, correlation between volume dynamic data, considered regional tolerance and systematic increase of marginal dose prescription (PPD) at each session was of essence to limit toxic dose dissipation to perilesional healthy tissue; in this, we used LQ model-based BED conversions plotted to EQD2-estimates and normal brain tissue tolerance, utilizing an α/β ratio of 2 (appendix 5). Although still a source of debate in the field of SRS and extreme hypofractionation (staged procedures included), we suggest that the LQ model remains a reliable tool for physical doses of up to 10 Gy per fraction; published analytical data based on large experimental compilation such as those from Brenner et al., seem to support this assumption [29]. Speckter et al. (2019) further explored the subject, identifying three different schools of thought [90]. The first views the LQ model as a reflection of adequate dose-response relationships at high doses, aligning predictive data with clinical outcomes [90]. The second believes that the LQ model grossly underestimates tumour control at high dose delivery. In this scenario, scholars suggest that apart from inducing DNA strand breaks and chromosomal aberrations as seen in more conventional doses, SRS enhances indirect cell death through vascular damage and antigen expression at doses greater than 8–12 Gy per fraction [90]. Conversely, the third school advocates for cell death overestimation when applying the model in extreme fractionation, as it does not give enough consideration to the reduction of sub-lethal damage from the conversion of sub-lethal to lethal damage [90]. In line with our view, the group concluded that, to some degree, the latter two antagonistic mechanisms may compensate each other [90]. Unavoidably, these considerations have repercussions on study setups, hence also on clinical praxis. In a study evaluating LTC and radionecrosis on patients receiving single fraction SRS vs. multifraction SRS, Minniti et al. adjusted LQ-plotted estimates to high doses [3]; using an α/β of 12, the BED of 27 Gy in 3 fractions was calculated at 40 Gy, corresponding to a single dose of approximately 22 Gy from adapted computations. The same group reported best incidence of local control and lower ARE in the hypofractionation arm; furthermore, important conclusions were drawn in relation to V_12Gy_ and V_18Gy_ ARE predictability [3], widely applied in clinical practice today [76]. Contrasting with the latter, Higuchi et al. conceived a staged therapy schedule of 10 Gy x 3 from standard 20 Gy single dose delivery utilising LQ-based calculations with no major deviations, ultimately reporting excellent outcome, including of neurological death avoidance [78]. In summary, considering the above dataset and the reliability of the LQ-model at lower α/β values (normal tissue response) [42], we submit to the reader that, in the case of our protocol, PPD-calculations can be safely applied with peripheral doses of up to10Gy, as it is aimed to spare healthy perilesional tissue from radiation-induced damage. Furthermore, regional tolerance is finely intertwined with iso-BED-estimations; it is widely accepted that the risk of ARE from regional cumulative doses of approximately 100 Gy (EQD2) is < 10% for recurring lesions [37, 38, 91, 92]. However, in accordance to our institutional experience, the latter cannot be indiscriminately applied to all parts of the brain anatomy, particularly highly eloquent/functional areas such as the thalamus, the hippocampus and the motor-sensor cortex, among others; in the context of our study, the above underwriting was selectively applied in accordance to neighboring OAR and the treating radiosurgeon’s experience and interpretation of regional radiotolerance. In a compensatory fashion, the systematic increase of PPD in relation to early signs of response at each stage to overcome possible issues of under-dosage in relation to the previous fraction has a rationale. Brown et al. analysed the relation between tumour control probability and BED in early-stage NSCLC patients undergoing radiotherapy with single fraction, 3–8 fractions and > 10 fractions [40]; the 3–8 fraction cohort was associated with higher TCP at BED > 100 Gy, with clear effects resulting from BED increments of just 10–20 Gy [40]. Furthermore, multiple studies have described the prognostic role of BED-based dosimetry in a range of histology portfolios, such as vestibular schwannomas and metastatic lesions; apart from factors innate to treatment schedule, planning, and delivery, it has been suggested that dose escalation and physical dose shifts (even within a 2 Gy range) may carry significant repercussions on perilesional organ toxicity and tumour ablation [35, 40, 93–96]. Extrapolated to RRR, a hypothetical dose increase from 8 Gy (BED = 40 Gy) to 9 Gy (BED = 49.5  Gy) at the margin (1 Gy physical dose difference, resulting in almost 10 Gy BED increment) is likely to play a determinant role on local control and toxicity, at least in theory [46, 47, 75, 80, 97]. Consequently, seeking to optimise cell death without compromising perilesional toxicity while calling to mind the novelty of this prescription approach, we opted for a cautious PPD increase of 0.5–1 Gy per fraction. Obviously, the approach has limited backup in terms of evidence and may well require development; however, the technique bears more latitude compared to subjective dose prescriptions in the face of convoluted lesional environment in routine praxis, such as volume, critical location and/or post-radiation relapsing. Complementarily, it is worth noticing the use of low prescription isodose lines (35–40%) in contrast with the usual higher isodose line (50% or higher). Although studies on the use of low prescription isodose remain scarce, the claim that a steepest dose fall-off is best achieved by utilizing the traditional 50–60% prescription isodose line does not apply to all cases [41], particularly in adaptive settings hinging on volume shift throughout treatment. In this study, the use of a lower isodose line allowed us to maximise the intratumoral V_10Gy_-coverage and optimise ‘hot-spot’ distribution in more solid areas of the tumour bed [41, 82, 98]. Despite this, our view on the matter remains fundamentally an institutional observation acquired on a limited number of patients and ought to be taken with caution, hence the need for prospective studies to confirm the validity of this procedural step.

The conception of a second prescription dose dynamically adapting to tumour volume changes between sessions, was also a novel aspect of treatment planning and requires further explanation. From a biological perspective, hypofractionation can be regarded as a collection of high dose-dependent interacting events likely to trigger ablation beyond the hallmarks of BED-estimates (generally set at 10 Gy per fraction or more) which includes reoxygenation, reperfusion, DNA-damage and endovascular cell apoptosis/vascular damage [9, 10, 21, 22, 27, 28, 38, 42, 48, 49, 85, 96, 97, 99–105]. Additionally, it is well known that the α/β ratio of tumours differ in accordance to histology [32]; moreover, due to possible molecular and genetic uncertainties likely to influence repair and sensitivity, the ratio may well vary among patients diagnosed with the same form of cancer [106]. Adding the current lack of consensus on the reliability of the LQ-model in extreme dose per fraction, the conception of a separate ablative prescription beyond BED-estimations seems rational. In a single centre retrospective study, Lesueur et al. reported the outcome of stereotactic radiation on 60 patients with 193 radioresistant BM < 3 cm (Cyber knife) [31]; according to LQ model-based estimates applying an α/β ratio of 2.5, SRS patients (median 20 Gy) received a median BED_2.5_ of 180 Gy (27% of the population) while those treated with hypofractionation, the BED_2.5_ was 122 Gy and 150 Gy for the 6 × 6 Gy (18%) and the 3 × 10 Gy (55%) schedule (median), respectively. Noticing that local control was grossly the same for all subgroups, the group concluded that the LQ model was not predictive of local control, emphasizing its weaknesses when prognosing tumour ablation; the same group also highlighted that, since LQ model cell kill is based on DNA strand breaks, it underestimates indirect cell death by devascularization and radiation-triggered immune responses in dose per fraction > 8–10 Gy [107]. Moreover, 10 Gy peripheral dose prescriptions are commonly used in staged procedures with good outcomes [78]. Therefore, aiming to modulate the above-described radiobiological cascades in a dose-volume manner, it made sense to conceive a second prescription dose structured on V_10Gy_ coverage (%), dynamically adapted to morphology changes at each fraction. The result was an average V_10Gy_ increase from 69 to 84% (GKRS 1–3) for radiosensitive lesions and 84–92% for radioresistant histology which we think may have a had a boosting ablative effect considering the results achieved in some of the patients; as reminder, lacking prospective data, this planning stage remains a theoretical approach. More details on the rationale applied here can be found in previous publications [4–7, 26]. Finally, the accelerated timeline of the technique, possibly the shortest among staged procedures, has intrinsic clinical advantages, particularly in more frail individuals where a 2–4-week delay between fractions may compromise treatment completion and hence inclusion. In the scope of pre-treatment evaluation (GKRS 2–3), a shorter inter-fraction interval leads unavoidably to closer monitoring, ultimately enhancing patient safety. From a radiobiological perspective, it can be argued that an accelerated timescale of 72-hour inter-fraction interval along with an ablative 10 Gy-volume based prescription, maximises the benefits of balancing local re-oxygenation and tumour vascular damage as discussed earlier on [21, 42, 106, 107]. Again, theoretically, a short schedule layout may also increase the window of inter-fraction cell repair while simultaneously decreasing the risk for tumoral repopulation [106].

#### Limitations

Several limitations arise from the workup and outcome of this study. Despite the positive results noted within the seven-year follow-up, the small number of patients with a rather wide histological heterogeneity was a limiting factor, ultimately compromising the level of evidence. Larger cohorts of patients with homogenic histology and similar clinical parameters are necessary to rationalise the dose-effect relationship at long-term, especially on local tumour control and neurological survival. In addition, the absence of a control group set on best supportive care or standard radiotherapeutic options prevented comparative validation. The results reported by Minnti et al., previously described in this paper, highlight this dilemma [3]. However, studies structured on homogenic groups with no access to other active therapeutic options may trigger ethical controversies, including patient randomisation for best management in spite of underlying elements advocating treatment such as the patient’s express wishes and fitness for therapy. Therefore, the use of control groups in such a hostile clinical environment becomes viable when active options are still applicable, which was not the case in our patient cohort. For instance, in a study of 3-stage GKRS for large BM, Yamamoto et al. used competing risk analysis to validate their results, which included a re-analysis of a previous study using the same technique [72]. Provided future studies on the subject, the aforementioned approach may well help overcome some of the limitations observed in this study. However, despite these obstacles, promising results have been shown from studies conducted without control groups [10, 78]. Unavoidably, the retrospective nature of this study was also a hindrance; hence, studies with a prospective workup are encouraged. Finally, although not a strong limitation here [108], future studies ought to consider using an α/β ratio of 2.5 for normal brain to remain consistent with the latest trend in the literature [108].

## Conclusion

Based on the results of this first retrospective study, we cautiously state that the use of concurrent and customised double prescription in adaptive accelerated settings proved to be feasible in terms of avoidance of neurological death, salvage of neurological function, acceptable rates of LTC, and low rates of symptomatic ARE in a group of patients with a poor prognosis and no possibility for active intervention. Despite being in its infancy, while confronted with extreme pre-treatment adverse factors, the procedure was able to match the outcome of some of the recent studies on the subject. Above all expectations, 2 patients were still alive almost 10 years post-treatment with no major sequelae despite initial poor prognosis. Although extracranial disease remains a source of concern in terms of patient selection and survival post-treatment, this study underlined the potential role of double prescription-based, adaptive radiosurgery in patients with precluded prognosis / survival, particularly in customised settings. However, the retrospective nature of the study and the restrained number of patients with variable histology included were obvious limitations. Therefore, prospective studies involving a larger number of patients with homogenic histology are warranted to validate the results of this study and further optimise the procedure.

## Electronic supplementary material

Below is the link to the electronic supplementary material.

Appendix 1: Case example - Dose prescription calculation (PPD and V10) - Brainstem

Appendix 2: Case example - Dose prescription calculation (PPD and V10)– Non-brainstem case

Appendix 3: Single-variable relations using cox regression analysis with hazard ratios (HR) and their 95% confidence intervals. The cox regressions were calculated independently with each variable versus the overall survival with censorship for survivors

Appendix 4: Kaplan-Meier Curve for Overall Survival

Appendix 5: Used BED-formula with derivates applied for PPD-conception (first prescription dose)

## Data Availability

No datasets were generated or analysed during the current study.

## Abbreviations

ARE: Adverse Radiation Effect
BED: Biological Effective Dose
BM: Brain Metastasis
CNS: Central Nervous System
EQD2: Equivalent Dose in 2 Gy
GKRS: Gamma Knife Radiosurgery
GTV: Gross Tumour Volume
HFSRS: Hypofractionated Stereotactic Radiosurgery
KPS: Karnofsky Performance Score
LGP: Leksell Gamma Knife Planning
LINAC: Linear Accelerator
LTC: Local Tumour Control
MRI: Magnetic Resonance Imaging
OS: Overall Survival
PET: Positron Emission Tomography
PFS: Progression Free Survival
PPD: Protective Peripheral Dose
RPA: Recursive Partitioning Analysis
SFSRS: Single Fraction Stereotactic Radiosurgery
WBRT: Whole Brain Radiation Therapy
WHO: World Health Organisation
