# Correction: Management of critically located brain metastases in patients with precluded survival using customised double-dose prescription-based, adaptive accelerated staged radiosurgery: a long-term retrospective analysis

**DOI:** 10.1186/s13014-025-02768-8

**Published:** 2026-01-22

**Authors:** G. Sinclair, H. Martin, C. M. Allison, M. A. Hatiboglu, H. Speckter, A. Fytagoridis

**Affiliations:** 1 Department of Neuroradiology, Karolinska University Hospital, Stockholm, Sweden; 2 Department of Neurosurgery, Karolinska University Hospital, Stockholm, Sweden; 3 Department of Neurosurgery, Bezmialem Vakif University Hospital, Istanbul, Turkey; 4 Department of Neurosurgery, Royal Victoria Infirmary, Newcastle-upon- Tyne, UK; 5 Department of Oncology, University Hospital Southampton NHS Foundation Trust, Southampton, UK; 6 Centro Gamma Knife Dominicano, CEDIMAT, Santo Domingo, Dominican Republic

**Correction: Radiat Oncol (2025) 20:120**

10.1186/s13014-025-02692-x

After publication of the article, it was brought to our attention that some author corrections were not well implemented in production, below is a list of corrections which have been implemented in the original article.

1. Keywords should be: Brain metastases, Radiosurgery, Gamma Knife, Linear accelerator, Prescription dose, Hypofractionated radiotherapy, Staged radiosurgery
2. Treatment protocol, under ‘second prescription’: Wrong (typo)- …8–10 GGy/fraction and (ii)…… Correct: …8–10 Gy/fraction and (ii)..
3. Treatment protocol, under ‘second prescription’: Typo: ‘Variables defining theintratumoral V_10Gy_ at GKRS 1–3:’. Correct: ‘Variables defining the intratumoral V_10Gy_ at GKRS 1–3:’
4. Typo/wrong: your staff included my instructions in the text! Wrong: ‘Sentence should start: From a statistical standpoint, Cox regression analysis was used to identify….’ Correct: ‘From a statistical standpoint, Cox regression analysis was used to identify….’
5. Treatment protocol, under ‘second prescription’: use ‘intratumoral’ throughout the text (not intra-tumoral)
6. Final version of Table 1 not uploaded
7. Multiple typos in Table 2 (final version not previously uploaded); the survival in months for case 1 and case 2 was rectified on the galley proof but not picked up by the system (no comment box showing): For Case 1 (breast histology) should read ‘117(alive)’ (not 93), For case 2 (Thymus histology) it should read ‘115(alive)’ (not 90). Case nr 7 – multiple typos in columns ‘met volume’, ‘first prescription’ and ‘second prescription’ (all compressed). Please check my original instructions
8. Multiple typos in Table 3.
9. Final version of Table 4 not uploaded
10. Discussion section: typo: ‘….substandard. KPS/RPA class ….’ should read ‘…substandard KPS/RPA class…’
11. Discussion section: typo: ‘….radiotherapy (7 out of 29 patients. Although….’ Correct: ‘.radiotherapy (7 out of 29 patients). Although.’.
12. Discussion section: Please check my previous instructions (asked to delete the appendix as reference here) Incorrect: ‘…24 and 36 months (Tables 3 and 4) (appendix).’ Correct: ….’24 and 36 months (Tables 3 and 4).’
13. Discussion section: Typo (repetition): ‘Another point worth mentioning is the fact is the fact that…’ Correct: ‘Another point worth mentioning is the fact that….’
14. Discussion section: Typo:’ .to’mimick’ the ablative effects of 18Gy single…’ Correct: ‘…to ‘mimic’ the ablative effects of 18Gy single…. ‘
15. Discussion section: Typo (repetition): ‘with clear effects resulting from BED increments of just 10–20 Gy [40], with clear effects resulting from BED increments of just 10–20 Gy [40].’ Correct: ‘….with clear effects resulting from BED increments of just 10–20 Gy [40]. Furthermore…. ‘
16. Discussion section: typo: ‘…resulting in almost 10 Gy ‘BEDincrement). Correct: ‘…resulting in almost 10 Gy BED increment)’
17. Limitations section: Sentence previously corrected yet not picked up by system (no comment box retained): Incorrect: ‘….ethical controversies, such as patient randomisation for best management in spite of underlying pro-treatment factors such as the patient’s express wishes and fitness for therapy’. Correct: ‘…ethical controversies, including patient randomisation for best management in spite of underlying pro-treatment factors such as the patient’s express wishes and fitness for therapy’.
18. Acknowledgement section: Typo: ‘writing and publishing of this paper.’ (double end period). Should read ‘writing and publishing of this paper.’
19. Supplementary information: ‘Track changed’ versions were published by mistake; clean versions to be updated in the original article
20. Abbreviation not corrected previously: Correct : ARE = Adverse Radiation Effect

The original publication has been updated. The publisher apologizes to the authors and readers for the inconvenience caused.

