# Supplementary material for: Management of critically located brain metastases in patients with precluded survival using customised double-dose prescription-based, adaptive accelerated staged radiosurgery: a long-term retrospective analysis
Source: Radiat Oncol. 2025 Aug 1;20:120. doi: 10.1186/s13014-025-02692-x (PMC12317634; doi:10.1186/s13014-025-02692-x)

Appendix 1

Case example - Dose prescription calculation (PPD and V_{10Gy}) - Brainstem Protocol

Variables

1. Location: Intrinsic metastatic lesion in the pons
2. Previous RT in the CNS: No (no annual dose decay)
3. Age: 64
4. KPS/RPA: 90-100 / 2
5. Histology: Breast cancer (radiosensitive: no need to adjust PPD at GKRS 1)
6. OAR: Entire brainstem
7. Tumour volume at GKRS 1: 5.2cc
8. Regional radiotolerance EQD2 to brainstem: 54Gy entire axis, 60Gy 1/3 of the brainstem.
9. α/β ratio used: 2

Two dose prescriptions required:

1. PPD (first prescription dose): Protection of perilesional tissue based on EQD2 estimates and BED iso-conversions

$$BED\ Gy = D\left(1 + \frac{d}{\alpha/\beta}\right)$$

2. V_{10Gy} conception and modulation (second prescription): Baseline isodose line for tumour ablation

1. **PPD calculation (GKRS 1 – 3)**

- BED max radiotolerance EQD2 (entire axis)

$$BED = 54\left(1 + \frac{2}{2}\right) = 108Gy$$

- BED max radiotolerance EQD2 (1/3 axis)

$$BED = 60\left(1 + \frac{2}{2}\right) = 120Gy$$

- Range of perilesional radiotolerance = 108 – 120Gy BED

- 3 fractions conversion for regional radiotolerance:

108 – 120Gy BED= 7Gy x 3 (BED 94.5 Gy) and 8Gy x 3 (BED=120Gy).

Prescription plan: PPD at GKRS 1 (estimated dose by average): 7.5Gy. PPD at GKRS 2-3 to be increased with 0.5-1Gy at each session if tumour volume reduction > 1cc and/or lesional necrotic changes

2. Conception and modulation of V_{10Gy}

- V_{10Gy} GKRS 1: to cover at least 70-80% of the tumour bed

- V_{10Gy} GKRS 2: to be increased under planning, when possible, without compromising the adjusted PPD

- V_{10Gy} GKRS 3: to be further increased under planning, when possible, without compromising the adjusted PPD.

Prescription plan: V_{10Gy} to be gradually increased at each session, at least 5 -10% from GKRS 1 to GKRS 3 (when possible).

Double-dose prescription outcome (per session and long-term follow-up):

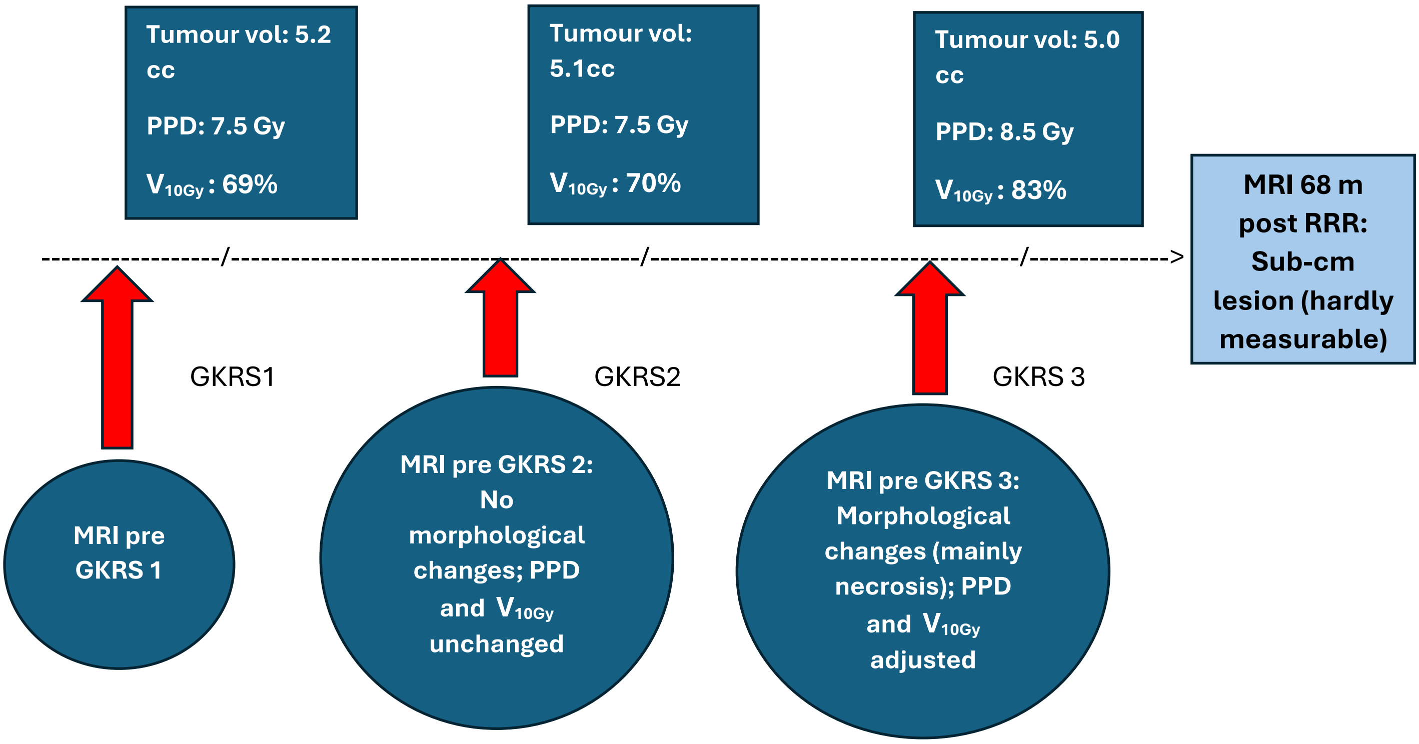

Supplement: Supplementary file 1 — Appendix 1: Case example - Dose prescription calculation (PPD and V10) - Brainstem [file 13014_2025_2692_MOESM1_ESM.pdf]
