# Supplementary material for: Management of critically located brain metastases in patients with precluded survival using customised double-dose prescription-based, adaptive accelerated staged radiosurgery: a long-term retrospective analysis
Source: Radiat Oncol. 2025 Aug 1;20:120. doi: 10.1186/s13014-025-02692-x (PMC12317634; doi:10.1186/s13014-025-02692-x)

Appendix 2

Case example - Dose prescription calculation (PPD and V_{10Gy}) – Outside-brainstem location protocol

Variables

1. Location: Frontoparietal region
2. Previous RT in the CNS: No (no annual dose decay)
3. Age: 56
4. KPS/RPA: 90 / 2
5. Histology: RCC (Highly radioresistant: PPD at GKRS 1 to be adjusted by adding 1Gy)
6. OAR: motor-sensor region
7. Tumour volume at GKRS 1: 12.0cc
8. Regional radiotolerance EQD2: 54-60Gy (as per institutional experience)
9. α/β ratio used: 2

Two dose prescriptions required:

1. PPD (first prescription dose): Protection of perilesional tissue based on regional EQD2 estimates and BED iso-conversions

$$BED\ Gy = D\left(1 + \frac{d}{\alpha/\beta}\right)$$

2. V_{10Gy} conception and modulation (second prescription): Baseline isodose line for tumour ablation

1. PPD calculation (GKRS 1 – 3):

- BED max radiotolerance based on EQD2 estimates (54Gy-60Gy): 108 – 120Gy

- 3 fractions conversion for perilesional radiotolerance:

108 Gy -120 Gy BED= 7Gy x 3 (BED 94.5 Gy) and 8Gy x 3 (BED=120Gy)

Prescription plan: based on BED data, preliminary PPD at GKRS 1 estimated at 7.5Gy (average dose); due to radioresistant histology, PPD further adjusted to 8.5Gy (7.5Gy +1Gy). PPD at GKRS 2-3 to be increased with 0.5-1Gy at each fraction if tumour volume reduction > 1cc and/or lesional necrotic changes

2. V_{10Gy} conception and modulation:

- V_{10Gy} GKRS 1: to cover at least >80% of the tumour bed

- V_{10Gy} GKRS 2: to be increased under planning, when possible, without compromising the adjusted PPD

- V_{10Gy} GKRS 3: to be further increased under planning, when possible, without compromising the adjusted PPD.

Prescription plan: V_{10Gy} to be gradually increased at each session, at least 5 -10% from GKRS 1 to GKRS 3 (when possible).

Double-dose prescription outcome (per session and long-term follow-up):

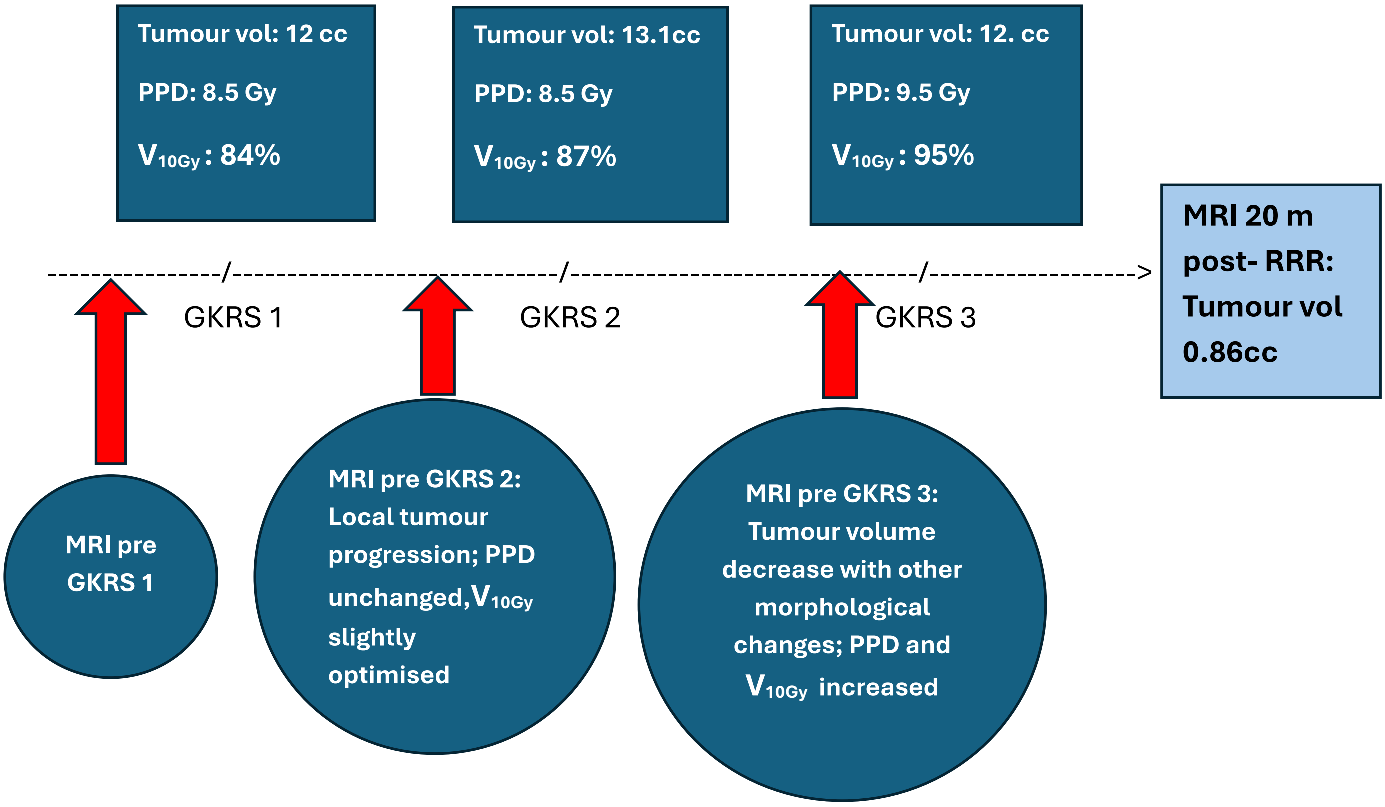

Supplement: Supplementary file 2 — Appendix 2: Case example - Dose prescription calculation (PPD and V10)– Non-brainstem case [file 13014_2025_2692_MOESM2_ESM.pdf]
