# Supplementary material for: Management of critically located brain metastases in patients with precluded survival using customised double-dose prescription-based, adaptive accelerated staged radiosurgery: a long-term retrospective analysis
Source: Radiat Oncol. 2025 Aug 1;20:120. doi: 10.1186/s13014-025-02692-x (PMC12317634; doi:10.1186/s13014-025-02692-x)
Supplement: Supplementary file 3 — Appendix 3: Single-variable relations using cox regression analysis with hazard ratios (HR) and their 95% confidence intervals. The cox regressions were calculated independently with each variable versus the overall survival with censorship for survivors [file 13014_2025_2692_MOESM3_ESM.pdf]

Appendix 3. Single-variable relations using cox regression analysis with hazard ratios (HR) and their 95% confidence intervals. The Cox regressions were calculated independently for each variable, with overall survival and censoring for survivors.

**Variable**	**HR**	**95% CI**		**p-value**
Age	1.047	1.007	1.088	0.020*
Total target volume	1.000	0.972	1.029	0.989
WBRT	0.729	0.293	1.813	0.496
Systemic therapies	0.789	0.363	1.713	0.548
KPS	0.939	0.903	0.977	0.002**
RPA	2.808	0.189	1.876	0.017*
