# Supplementary figures and images for: Management of critically located brain metastases in patients with precluded survival using customised double-dose prescription-based, adaptive accelerated staged radiosurgery: a long-term retrospective analysis
Source: Radiat Oncol. 2025 Aug 1;20:120. doi: 10.1186/s13014-025-02692-x (PMC12317634; doi:10.1186/s13014-025-02692-x)

#### Appendix 4 – Kaplan-Meier Curve for Overall Survival

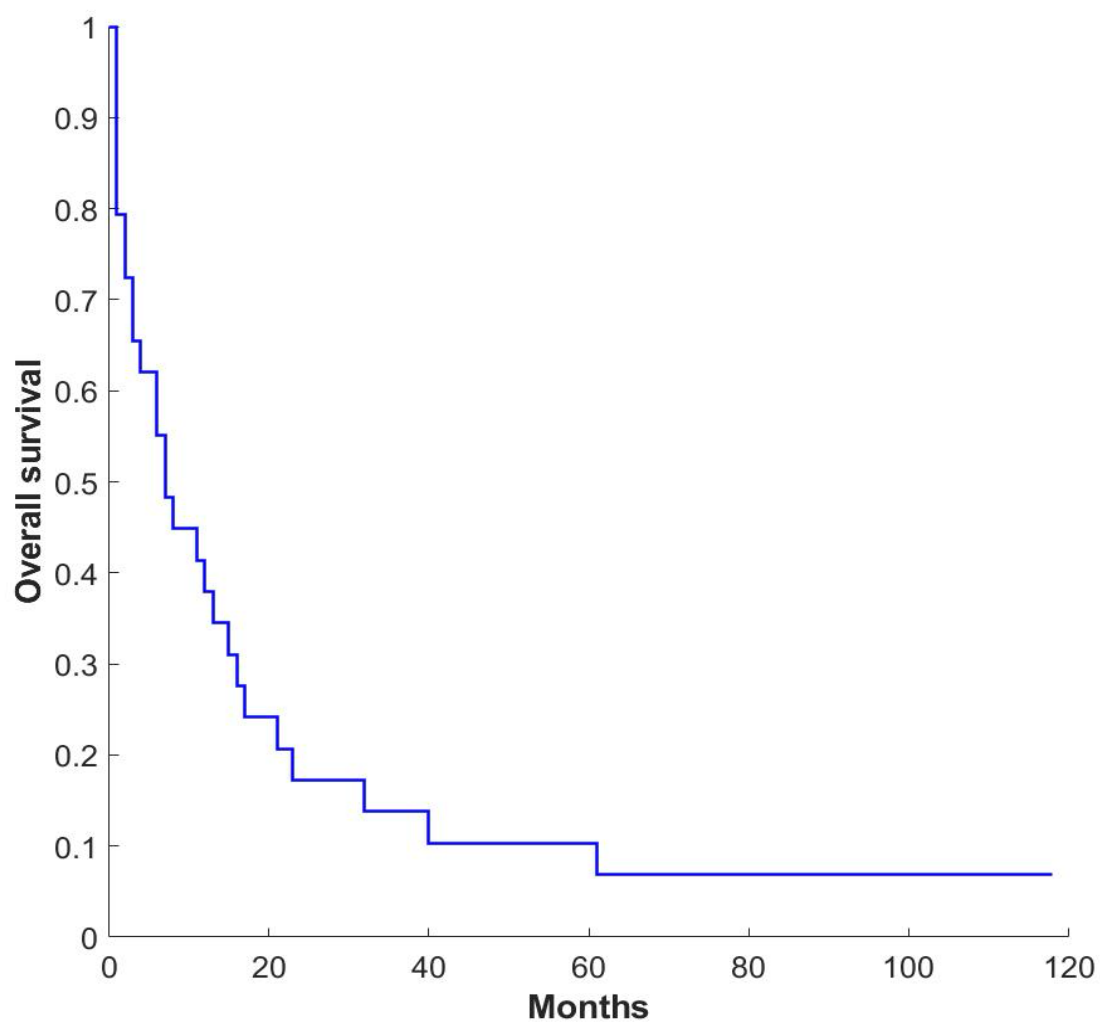

Supplement: Supplementary file 4 — Appendix 4: Kaplan-Meier Curve for Overall Survival [file 13014_2025_2692_MOESM4_ESM.pdf]
