# Supplementary material for: Management of critically located brain metastases in patients with precluded survival using customised double-dose prescription-based, adaptive accelerated staged radiosurgery: a long-term retrospective analysis
Source: Radiat Oncol. 2025 Aug 1;20:120. doi: 10.1186/s13014-025-02692-x (PMC12317634; doi:10.1186/s13014-025-02692-x)
Supplement: Supplementary file 5 — Appendix 5: Used BED-formula with derivates applied for PPD-conception (first prescription dose) [file 13014_2025_2692_MOESM5_ESM.pdf]

Biologically Effective Dose (BED)

Survival Fraction: $S.F. = e^{-E} = e^{-(\alpha D+\beta D^2)}$

$\Downarrow$

Effect (cell death): $E = nd(\alpha + \beta d)$

$\Downarrow$

Biologically Effective Dose: $BED = E/\alpha = nd(1+d/\alpha/\beta)$

Total dose D=nd; d=dose per fraction

Appendix 5: Used BED-formula with derivates applied for PPD-conception (first prescription dose).
